# Voice Disorder Detection via an m-Health System: Design and Results of a Clinical Study to Evaluate Vox4Health

**DOI:** 10.1155/2018/8193694

**Published:** 2018-08-08

**Authors:** Ugo Cesari, Giuseppe De Pietro, Elio Marciano, Ciro Niri, Giovanna Sannino, Laura Verde

**Affiliations:** ^1^Department of Otorhinolaryngology, University Hospital (Policlinico) Federico II of Naples, Via S. Pansini 5, Naples, Italy; ^2^Institute of High Performance Computing and Networking (ICAR-CNR), Via Pietro Castellino 111, Naples, Italy; ^3^Area of Audiology, Department of Neurosciences, Reproductive and Odontostomatological Sciences, University of Naples Federico II, Via S. Pansini 5, Naples, Italy; ^4^Independent Doctor Surgeon, Specialized in Audiology and Phoniatrics, Naples, Italy; ^5^Department of Engineering, University of Naples “Parthenope”, Centro Direzionale di Napoli, Isola C4, Naples, Italy

## Abstract

**Objectives:**

The current study presents a clinical evaluation of Vox4Health, an m-health system able to estimate the possible presence of a voice disorder by calculating and analyzing the main acoustic measures required for the acoustic analysis, namely, the Fundamental Frequency, jitter, shimmer, and Harmonic to Noise Ratio. The acoustic analysis is an objective, effective, and noninvasive tool used in clinical practice to perform a quantitative evaluation of voice quality.

**Materials and Methods:**

A clinical study was carried out in collaboration with medical staff of the University of Naples Federico II. 208 volunteers were recruited (mean age, 44.2 ± 13.9 years), 58 healthy subjects (mean age, 36.7 ± 13.3 years) and 150 pathological ones (mean age, 47 ± 13.1 years). The evaluation of Vox4Health was made in terms of classification performance, i.e., sensitivity, specificity, and accuracy, by using a rule-based algorithm that considers the most characteristic acoustic parameters to classify if the voice is healthy or pathological. The performance has been compared with that achieved by using Praat, one of the most commonly used tools in clinical practice.

**Results:**

Using a rule-based algorithm, the best accuracy in the detection of voice disorders, 72.6%, was obtained by using the jitter or shimmer value. Moreover, the best sensitivity is about 96% and it was always obtained by using jitter. Finally, the best specificity was achieved by using the Fundamental Frequency and it is equal to 56.9%. Additionally, in order to improve the classification accuracy of the next version of the Vox4Health app, an evaluation by using machine learning techniques was conducted. We performed some preliminary tests adopting different machine learning techniques able to classify the voice as healthy or pathological. The best accuracy (77.4%) was obtained by the Logistic Model Tree algorithm, while the best sensitivity (99.3%) was achieved using the Support Vector Machine. Finally, Instance-based Learning performed the best specificity (36.2%).

**Conclusions:**

Considering the achieved accuracy, Vox4Health has been considered by the medical experts as a “good screening tool” for the detection of voice disorders in its current version. However, this accuracy is improved when machine learning classifiers are considered rather than the rule-based algorithm.

## 1. Introduction

Voice signals are sounds produced by air pressure vibrations exhaled from the lungs and modulated and shaped by the vibrations of the vocal folds and the resonance of the vocal tract. The physiological process that leads to the production of the voice involves several structures, such as

- the* respiratory system*, the main component that influences the intensity of the voice thanks to modulation of an expiratory flow with a variable pressure below the vocal folds;
- the* larynx*, the cornerstone of the production of the voice, especially through the vocal folds whose vibration determines the sound;
- the* vocal tract*, constituted by the pharynx and nasal and paranasal cavities, responsible for sonority, changes in the timbre, and resonance of the laryngeal sounds.

In addition, there are the* auditory* and* central nervous* systems. The former plays an important role in regulating the intensity of the voice interacting with the central nervous system that participates in the management of several mechanisms involved in the production of the voice, such as breathing or pneumophonic coordination [1].

An alteration, functional or morphological, of this mechanism can cause a degradation of the quality and intensity of the voice, due to specific diseases. Dysphonia, the medical word that indicates a voice disorder, affects about 10% of the population at least once in their lifetime [2] and the percentage increases to 50% among voice professionals, such as teachers or singers. Vocal abuse or incorrect lifestyle habits, such as smoking or alcohol abuse, constitute risk factors for the development of the disorder.

For the clinical evaluation of the voice several medical examinations are required. In accordance with the SIFEL (Società Italiana di Foniatria e Logopedia, the Italian Society of Logopedics and Phoniatrics) protocol [3], the Italian medical protocol approved by the Committee for Phoniatrics of the European Society of Laryngology, it is necessary to perform

- a direct observation of the vocal tract through a laryngoscope;
- a self-assessment of the patient's state of health;
- an accurate anamnestic investigation;
- an objective estimation of the characteristic parameters required for the acoustic analysis.

This latter aims to quantify and characterize a voice quality in a noninvasive manner, processing a voice signal of a vocalization of the vowel /a/ of five seconds in length. Several studies existing in literature, such as [4, 5], have demonstrated the relationship between the acoustic measures and laryngeal functionality.

The main acoustic measures are

- the Fundamental Frequency (F_0_) that represents the vibration of the vocal folds;
- perturbation measurements that consist in an examination of the variability of the glottal cycles in terms of frequency (jitter) and amplitude (shimmer);
- noise measures, such as the Harmonic to Noise Ratio (HNR) that evaluates the presence of noise in the voice signal.

Smart computer-based systems can be used as an adequate support for the assessment and detection of voice disorders.

Vox4Health is an m-health solution able to estimate the main acoustic parameters [6]. It is capable of acquiring the user's vocal signal by using the microphone of a mobile device, of elaborating this signal by calculating in real time the acoustic parameters, and of identifying any possible alteration due to the presence of a voice disorder.

The aim of this clinical study is to evaluate its accuracy in the detection of voice disorders. An experimental phase was carried out in collaboration with the specialized medical staff of the University of Naples Federico II. We developed an appropriate trial protocol, a detailed document, realized in accordance with the guidelines of medical specialists, the SPIRIT (Standard Protocol Items: Recommendations for Interventional Trials) 2013 Statement [7], and the SIFEL protocol, to define all the procedures to be followed for the evaluation of Vox4Health.

## 2. Related Work

Several systems are able to perform the acoustic analysis. Some of them are widely used in clinical practice, such as the Multidimensional Voice Program (MDVP) [8] or Praat [9, 10]. The former was developed by the Computerized Speech Lab (Kay Elemetrics Corporation, Lincoln Park, NJ, USA) and is currently one of the most commonly used and cited acoustic analysis software packages in literature. The latter, Praat (its name corresponding to the imperative form of “praaten”, “to speak” in Dutch), was realized by Paul Boersma and David Weenink of the Phonetic Sciences Department of the University of Amsterdam. These systems are capable of estimating several acoustic parameters useful for voice assessment, such as F_0_, jitter, shimmer, HNR, and the percentage of unvoiced segments. It is important to note that these systems are limited to estimating these parameters while their interpretation, such as if they are indexes of possible laryngeal alterations, is the responsibility of medical specialists.

In the scientific literature other computer systems have been presented, such as BioVoice, a solution proposed by Manfredi et al. [11]. It is able to evaluate several voice indexes, such as the F_0_, jitter, Relative Average Pertubation (RAP), and Adaptive Normalized Noise Energy (ANNE), to analyze pathological adult voices. The proposed approach was tested using three parameters, i.e., jitter, RAP, and ANNE, on a very limited set of only 15 patients suffering from cysts and polyps and 9 healthy subjects.

The Voice Analysis and Screening System (VASS) [12] is another computer system for acoustic analysis aimed at distinguishing pathological voices from healthy ones. This software calculates some widely used acoustic parameters and proposes two new indexes, the Turbulent Noise Index (TNI) and Normalized First Harmonic Energy (NFHE).

Hossain et al. [13] introduced a smart healthcare monitoring framework useful to voice pathology detection. Two types of input signals were used, the voice signal acquired by a recording device or a smartphone and an electroglottographic (EGG) signal captured by an EGG device. Local features from the voice signal and the shape and cepstral features from the EGG signal are extracted in the cloud. Using these features, the GMM-based classifier outputs a decision on the pathology detection.

It is important to note that most of the systems here reported cannot be considered as a personal and portable instrument, because they have been developed as desktop-based applications. The use of mobile devices, instead, can be a rapid and valid support to improve patient care, motivating individuals to obtain and analyze their health data, and, consequently, increasing interest in an underestimated pathology like dysphonia.

There are a few numbers of m-health systems capable of achieving an acoustic analysis. OperaVox [14] is an iOS-based app which estimates several voice parameters, including the F_0_ and perturbation measures (jitter and shimmer). One hundred voice samples were considered to evaluate the degree of agreement between OperaVox and MDVP, limiting to comparing the values of estimated parameters and not evaluating its capability of voice disorders detection. The signals were selected from among volunteers and patients at the Royal National Throat Nose and Ear Hospital, London. A wide range of voice disorders were included: muscle tension dysphonia, laryngopharyngeal reflux, vocal fold paralysis, spasmodic dysphonia, sulcus vocalis, and vocal fold lesions. The experimental tests showed that the performance of OperaVox is comparable to that of MDVP mainly in terms of measuring the F_0_, jitter, and shimmer of the voice, while the Noise to Harmonic Ratio (NHR) measurement shows a major difference.

Van Leer et al. [15] developed a mobile application for iOS devices designed to estimate the F_0_, jitter, and Cepstral Peak Prominence (CPP). In this preliminary study, only fourteen individuals, suffering from a variety of voice disorders, were recruited at the University of Cincinnati Voice and Swallowing Clinic limiting to test the usability of the proposed m-health solution.

It is important to note that the presented software solutions for the acoustic analysis use different algorithms for the calculation of the voice characteristic parameters, namely, the F_0_, jitter, shimmer, and HNR. There are no standard algorithms in literature for their estimation and this influences the classification accuracy of each system.

It is possible, for example, to estimate the F_0_ by using the Spectral Analysis [16], the Hilbert-Huang transform [17], the Robust Algorithm for Pitch Tracking (RAPT) [18], or the autocorrelation method [19]. In particular, this latter has been used by several systems, such as MDVP, Praat, and OperaVox. BioVoice, instead, estimates F_0_ by using a two-step procedure based on Simple Inverse Filter Tracking (SIFT) [20] and Average Magnitude Difference Function (AMDF) [19]. Vox4Health uses a personalized methodology described in [21], which we have designed and developed.

Additionally, the different methods used for calculating the F_0_ influence the measurements of jitter and shimmer, since their calculation is directly linked to the F_0_ value, although the main systems use the same characteristic formulas reported in the following section. Moreover, the HNR can be estimated using different methods, such as de Krom's algorithm or d'Alessandro's algorithm [22], in addition to using an autocorrelation approach as performed by Praat. Vox4Health uses de Krom's algorithm.

## 3. Vox4Health

**Figure 1:**
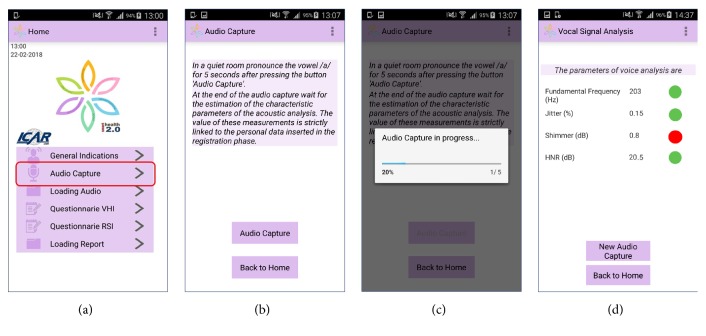
Screenshots of Vox4Health for the voice signal analysis procedure: (a) home screenshot, where the user must select the “*Audio Capture*” functionality to perform the voice acquisition; ((b) and (c)) Audio Capture screenshots, necessary to acquire the voice signal; and (d) voice signal analysis screenshot, where the estimates of characteristic acoustic parameters are shown.

Vox4Health is an Android application developed using the Java Programming Language through the use of Eclipse IDE and the Android Software Developer Kit (SDK). This app provides several functionalities, both to provide information about dysphonia and preventive healthy lifestyle behaviors and to complete specific self-assessment questionnaires required by the SIFEL protocol. The main functionality is to acquire the user's voice signal by using the microphone of a mobile device, such as a smartphone or tablet, to elaborate this signal (the vocalization of the vowel /a/ of five seconds in length), and to estimate in real time the characteristic parameters required by the SIFEL protocol, useful to identify possible alterations to the laryngeal tract. Finally, the estimation and evaluation of these parameters are shown to the user, as shown in Figure 1. In the current version of the app, a rules-based evaluation, specified in the Materials and Methods, of each parameter is implemented.

In detail, the app version evaluated in this clinical study is an improvement of the version described in [6]. In fact, the proposed solution estimates not only the F_0,_ with a methodology improved from that implemented in our previous version, but also three other specific parameters required by the clinical protocol. The choice of how many and which parameters to estimate and their unit of measurement was discussed and agreed with the medical specialists involved in this study. The acoustic parameters estimated are as follows:

(i) ***Fundamental Frequency (F***_0_**)**: this constitutes an important index of laryngeal function since it represents the rate of vibration of the vocal folds. The F_0_ is estimated with the methodology described in [21], an optimization of the Yin algorithm [23], that takes into account two of the main factors that influence the F_0_, namely, the gender and age of the subject.
(ii) ***Jitter***: this indicates the changes in the F_0_ cycle-to-cycle, representing the instabilities of the vocal folds. It is estimated, as indicated in [24], as the average difference between consecutive periods divided by the average period and expressed as a percentage, represented in
  (1) $$\begin{array}{c} Jitter\left(\;\%\;\right)=\frac{\left(1/\left(N-1\right)\right)\sum_{i=1}^{N-1}\left|T_{i}-T_{i+1}\right|}{\left(1/N\right)\sum_{i=1}^{N}T_{i}} \end{array}$$
where T_i_ are consecutive periods and N is the number of extracted F_0_ periods.
(iii) ***Shimmer***: this denotes the instabilities of the vocal folds quantifying the changes in amplitude cycle-to-cycle. It is expressed in decibels (dB) and calculated, according to [24], as indicated in (2), which is the average absolute base 10 logarithm of the difference between the amplitudes of consecutive periods, multiplied by 20:
  (2) $$\begin{array}{c} Shimmer\left(\;dB\;\right)=\frac{1}{N-1}\overset{N-1}{\underset{i=1}{\sum }}\left|\;20\log\left(\;\frac{A_{i+1}}{A_{i}}\;\right)\;\right| \end{array}$$
where A_i_ are the extracted peak-to-peak amplitudes and N is the number of extracted F_0_ periods.
(iv) ***Harmonic to Noise Ratio (HNR)***: this indicates the presence of noise in the signal due to an incomplete vocal fold closure, typical of some voice disorders. It is expressed in dBs and estimated according to de Krom's algorithm [22].

In our preliminary pilot study described in [25], the usability and user satisfaction of Vox4Health were evaluated. Here a previous version of the app was tested using two well-known usability test questionnaires, the System Usability Scale (SUS) [26] and the User Experience Questionnaire (UEQ) [27]. The first is a simple, reliable, and Likert scale tool able to provide an evaluation about the usability of the m-health system. UEQ, instead, assesses six basic aspects of the app: the attractiveness, perspicuity, dependability, efficiency, novelty, and stimulation. A pilot study was conducted in two regions of Italy, Campania and Calabria. At the end of this study, the users were encouraged to discuss any difficulties encountered using this app, the perceived quality of the information provided, and any preferences for different features. Based on these considerations, we have improved our solution and we have tested in the current study its reliability to estimate the presence of voice disorders analyzing a voice signal.

The Vox4Health app has been installed and tested on several Android devices, such as Mediacom Phone Pad duo, Samsung S4, Asus Zenfone 3, LG l70, One Plus One, Samsung S5, Samsung Galaxy Nexus, Samsung S6, and Huawei Mate 10 Lite. The operative system versions of the tested devices range from Android 4.1 to Android 8.0. The sampling rate of the recordings, processed to extract the characteristic acoustic parameters, is fixed in the app to a value of 8000 Hz, just as the range of measurements is similarly fixed.

## 4. Design of the Clinical Study

The conducted clinical study has been performed following a specific trial protocol approved by the Federico II University Ethics Committee. This protocol was developed in accordance with the guidelines of the SPIRIT 2013 [7] and SIFEL protocol and evidence from medical staff involved in the study.

The adopted protocol consists of several sections. The* Administrative Information* section, where all the administrative information is reported, such as the title that identifies the trial design and the roles and responsibilities of the contributors to the process. In the* Methods* section, all the plans to be followed during the clinical study are indicated, such as the period and study setting, the recruitment and eligibility criteria for the participants, and the procedures for the collection and evaluation of the data. The plans for the ethical approval of the trial process are, instead, defined in the* Ethics and Dissemination* section, where there are, also, the behavior norms and rules of the participants indicated in the informed consent and the processing of the personal data specified in the appropriate form. Finally, all documents are contained in the* Appendix *section, such as the informed consent, authorization for the processing of personal data, the information sheet for the process, and the anamnestic form. In detail, the procedures for the evaluation of Vox4Health are divided into several phases, as indicated in Table 1.

## 5. Materials and Methods

### 5.1. Participants

The clinical study started on May 16, 2016, and ended on May 15, 2017. The ambulatory surgeries of Phoniatrics and Videolaryngoscopy of the Hospital University of Naples “Federico II” and the medical room of the “Institute of High Performance Computing and Networking (ICAR-CNR)” were selected as the locations where the trial was conducted. People who met the inclusion criteria, those who had an age between 18 and 70 years and who were able to follow all phases of the clinical study, were invited to participate in the process. Subjects under 18 and over 70 or with diseases, such as colds or upper respiratory tract infections, or neurological disorders were excluded.

We recruited 208 volunteers, 58 with a healthy voice (21 males and 37 females) and 150 with a pathological voice (52 males and 98 females), suffering from several different voice disorders. The average age of the subjects involved is about 40 years, both for the women and men, while people with an age between 40 and 60 years represent the category of subjects that suffer the most from voice disorders. Some of these subjects have been diagnosed with a dysphonia by the medical experts, so we can divide the voice signals into four groups, one related to healthy voices and other three relating to three specific types of diseases:

- Hyperkinetic dysphonia (70 subjects, 47 women and 23 men)
- Hypokinetic dysphonia (41 subjects, 32 women and 9 men)
- Reflux laryngitis (39 subjects, 19 women and 20 men).

The first disorder is characterized by a striving and shrieking voice due to a muscular hypercontraction of the pneumophonic apparatus. The incomplete closure of the vocal folds causes, instead, a weak and breathless voice in patients suffering from hypokinetic dysphonia. Finally, a chronic hoarseness is the most common symptom of reflux laryngitis.

In Table 2, we have reported the number of collected voice samples, specifying how many voices we fall within each category (healthy or pathological), recording this information also by gender. Additionally, we have calculated the percentage for each category compared to the whole dataset.

The completion of two self-perception questionnaires, the Voice Handicap Index (VHI) and Reflux Symptom Index (RSI), was required by all participants.

The first questionnaire, the VHI, through the completion of 30 questions, evaluates the self-perception of impairment due to a voice disorder. Each question is assigned a score based on the severity of the symptom; the overall score allows a classification of the self-perception of disorder as mild, moderate, or severe. The obtained results in our study are shown in Table 3, where we indicate for each category of subjects the* VHI score*, necessary to classify the severity of the symptoms, the number of subjects that achieved the considered results, and the percentage (%) of subjects calculated compared to the whole dataset.

The RSI, instead, is a questionnaire to provide a self-perception for the assessment of laryngopharyngeal reflux, a risk factor for voice disorders. Also, in this case, each question is assigned a score from 0 (no problem) to 5 (severe problem). An overall score higher than 13 is considered abnormal; that is, the person has perceived symptoms of laryngopharyngeal reflux. We have summarized in Table 4 the obtained results, indicating for each category of subjects the* RSI score*, necessary to classify the severity of symptoms, the number of subjects that achieved the considered results, and the percentage (%) of subjects calculated compared to the whole dataset.

It is important to note that although the VHI and RSI evaluations are required by the SIFEL protocol, they are not used in clinical practice for the diagnosis. In fact, medical experts detect voice disorders through the objective evaluation provided by the acoustic analysis and through the direct vision of the vocal folds by performing the laryngoscopy. The VHI, as also the RSI, is not considered when making the diagnosis. In the same way, Vox4Health does not use these indexes to classify the voice as healthy or pathological. Therefore, the number of abnormal VHI results is not a source of bias, because the voices were classified considering only the estimations of the acoustic parameters.

However, these indexes are the demonstration that people who may suffer from dysphonia often underestimate its symptoms and therefore delay consulting a speech therapist for accurate voice assessment and treatment. A sore throat or a lowering of the voice are often underrated and not carefully treated, as indicated by the medical experience of the specialists involved in our study.

### 5.2. Analysis

To classify a voice as pathological or healthy, we evaluated the four voice features estimated by the app, namely, the F_0_, jitter, shimmer, and HNR, by using IF/THEN rules, and then we compared the results obtained with those achieved by Praat, one of main systems currently used in clinical practice, by using the same IF/THEN rules.

Both systems, Vox4Health and Praat, are able to estimate the characteristic parameters required by the acoustic analysis, although they use different algorithms to achieve that objective. In fact, for the estimation of the F_0_ value Vox4Health uses a personalized methodology described in [21], which we have designed and developed. Moreover, for the HNR value, Vox4Health uses de Krom's algorithm [22]. On the other hand, these two acoustic parameters (F_0_ and HNR) are estimated by Praat using the autocorrelation method [28]. The jitter and shimmer are, instead, estimated by both systems using the same formulas, as reported in the* Vox4Health*. The different methods for calculating F_0_ directly influence the measurements of jitter and shimmer, since their calculation is linked to the F_0_ value.

Additionally, it is important to note that Praat only calculates the main characteristic parameters but does not provide any suggestion or interpretation of these parameters (it does not classify if a voice is pathological or not). Vox4Health, instead, provides this interpretation by showing to the user a green circle if the value is within the healthy range and a red one in the other case. Moreover, another difference between the two systems is that Vox4Health is an m-health solution, portable and accessible anywhere and at any time from a mobile device, such as a smartphone or tablet. Praat, instead, is a desktop-based application and it is not accessible by a mobile system.

To identify the presence or not of a pathology it is possible to use a rule-based approach: the estimated parameters are evaluated according to IF/ELSE rules, comparing the values obtained with a fixed healthy range. Unfortunately, for these parameters, a standard healthy range does not exist [29], due to the dependence of some acoustic parameters on deterministic physiological factors. In this study, the healthy ranges necessary to perform the rule-based analysis were chosen in accordance with the indications of the medical specialists involved in the project and the main studies existing in literature, as indicated in the following subsection.

Details about the rule-based analysis are specified in the following subsections.

*Rule-Based Analysis*. The values obtained for each parameter were compared with a fixed healthy range of values applying the following IF/THEN rules to evaluate the ability of each acoustic parameter to identify possible alterations, indices of a pathological voice:

 
**IF** (estimated value of acoustic parameter is within the healthy range)
   
**THEN** Voice classified as* healthy*
 
**ELSE**
    Voice classified as* pathological*

The determination of the healthy ranges, shown in Table 5, was made in accordance with the indications of the medical specialists involved in the project and the main studies existing in literature. In detail, we have considered the healthy ranges indicated in [28–30] for jitter, shimmer, and HNR. Meanwhile there is no standard healthy range for F_0_, because, as mentioned previously, this is influenced by several factors, and so it is difficult to define a healthy range.

In one of our previous studies, we used the ranges provided by the medical experts from the Department of Otorhinolaryngology at the “University Magna Graecia” of Catanzaro, as indicated in [6]. However, deepening our studies, also in collaboration with the medical team of the University Hospital (Policlinico) Federico II of Naples, we understood that the healthy and pathological F_0_ ranges needed to be improved. For this reason, we have conducted a new more in-depth study in the scientific literature about this issue, and, based on the considerations contained in [31–33], we have suggested adopting different ranges from those indicated in [6]. The new healthy range used was calculated considering the mean and standard deviation values of the F_0_ indicated in these studies [31–33].

To compare the performance of Vox4Health and Praat, the same voice samples were processed by both systems and evaluated considering the same feature ranges to detect a voice as healthy or pathological.

## 6. Results and Discussion

The performance of Vox4Health was evaluated in terms of reliability and portability, that is, the capability of our system to achieve stable and consistent results for repeatable tests, evaluating the variations of each acoustic parameter, namely, F_0_, jitter, shimmer, and HNR, between several measurements. Additionally, we conducted a series of tests to evaluate the classification performance to discriminate between a pathological and a healthy voice. In the following subsections, the details of the performed analyses are explained.

### 6.1. Reliability Analysis

To evaluate the reliability of the app in the calculation of the acoustic parameters, we conducted a series of tests where we collected several voice recordings. Two voice signal acquisitions for each enrolled subject were recorded.

In 14% of cases (29 voices/208 = 0.139), the acoustic parameters calculated by the Vox4Health app diverge, in terms of classification results, between the different voice signals of the same user. This means that, in 14% of cases, one voice signal was classified as healthy and the other one of the same user was classified as pathological. However, it is extremely important to highlight that all these cases are due to the presence of a clearly audible noise in the files, such as the user coughs or laughs during the recording, or not completing correctly the vocalization, such as the signal having duration of less than 5 seconds.

Therefore, if we exclude the corrupted files from the reliability evaluation, the Vox4Health app is able to achieve the same classification result in the repeated signal acquisition. Please note that this means that the classification of the voice is exactly the same. However, the numerical values of the calculated acoustic parameters are not exactly the same because the signals from which they are calculated are not identical. In Table 6, we have reported, for the sake of brevity, only the values relative to a subset of enrolled subjects.

### 6.2. Portability Analysis

In order to demonstrate that the classification results do not change among the different Android devices and versions, we performed some tests in a quiet room where the same audio file was reproduced and acquired by four different devices, placed at the same distance from the audio file source: a Samsung S4 (Android version 5.0.1), a Huawei Mate 10 Lite (Android version 7.0.0), an Asus Zenfone 3 (Android version 8.0.0), and a One Plus One (Android version 8.0.0). As shown in Tables 7 and 8, the numerical values calculated by the devices are very similar and the classification results are exactly the same. Therefore, no mismatching cases were observed in terms of classification results. By evaluating the classification performance using different devices, it is possible to confirm that our system is stable and portable.

### 6.3. Classification Analysis

In this subsection, the results in terms of classification are presented. These were evaluated by means of an assessment of the* True Positives (TP), True Negatives (TN), False Positives (FP), *and* False Negatives (FN)*. The first group, the TPs, indicates cases when the voice sample is pathological and the acoustic parameter estimation recognizes this; the second, the TNs, indicates cases when the voice sample is healthy and the acoustic parameter estimation recognizes this; the third, the FPs, indicates cases when the voice sample is healthy and the acoustic parameter estimation regards it as pathological; and, finally, the fourth group, the FNs, indicates cases when the voice sample is pathological and the acoustic parameter estimation regards it as healthy.

The performances were estimated in terms of the following:

(i) ***Accuracy***: the percentage of samples classified correctly, calculated as indicated in
  (3) $$\begin{array}{c} Accuracy=\frac{TP+TN}{TP+TN+FP+FN} \end{array}$$
(ii) ***Sensitivity***: the system's ability to correctly classify a voice sample as diseased, expressed as
  (4) $$\begin{array}{c} Sensitivity=\frac{TP}{TP+FN} \end{array}$$
(iii) ***Specificity***: the system's ability to correctly classify a voice sample as healthy, calculated with
  (5) $$\begin{array}{c} Specificity=\frac{TN}{TN+FP}. \end{array}$$

*Rule-Based Analysis Results*. The results achieved from the comparison between Vox4Health and Praat by using the rule-based analysis are shown in Table 9.

The results obtained show that the proposed system provides a better accuracy in discriminating pathological voices from healthy ones, evaluating the F_0_, jitter, and shimmer better than Praat. In particular, instabilities of the vocal folds, typical of pathological voices, are well identified by the perturbations measures (jitter and shimmer), as indicated by the values of sensitivity, 96.0% for jitter and 89.3% for shimmer. Unfortunately, this instability also characterizes healthy voices, influencing the specificity obtained with the proposed m-health system. Praat, instead, has a better capability to classify correctly a voice sample as healthy, the specificity results obtained for F_0_, jitter, and shimmer being higher than those achieved with Vox4Health. However, the better specificity obtained in estimating the HNR was achieved using Vox4Health (51.7% versus 41.4%), with the accuracy values comparable between the two systems (61.1% for Vox4Health and 63.0% for Praat).

The voice disorders are distinguished in three groups: hyperkinetic or hypokinetic dysphonia and reflux laryngitis. The results achieved for these different pathologies are shown in Tables 10, 11, and 12. These results indicate that hyperkinetic dysphonia is well detected using Vox4Health, the accuracy achieved in estimating all acoustic parameters being higher than that achieved by Praat, a result confirmed when the sensitivity percentages are considered. However, for subjects suffering from hypokinetic dysphonia Praat provides a better accuracy than the proposed system, although the high sensitivity values indicate that the proposed system has a lower number of false negatives, which means that fewer pathological voices are erroneously evaluated as healthy. Additionally, considering subjects suffering from reflux laryngitis, the obtained sensitivities are higher than the values achieved with Praat for the F_0_, jitter, and shimmer. The HNR sensitivity obtained with Praat is better than that achieved with Vox4Health, but the percentages achieved for accuracy and specificity by Vox4Health are higher than those of Praat.

A possible reason for the different accuracy classification obtained by the two systems is due to use of different algorithms for the estimation of these parameters and the consequent different estimated values. As already mentioned in the subsection Analysis, Vox4Health and Praat use different algorithms to estimate these parameters, influencing the capability of correctly classifying a voice signal.

*Machine Learning Analysis.* To improve the classification accuracy obtained with the rule-based detection algorithm we have studied the use of some machine learning (ML) techniques. The idea is to identify the algorithm that obtains the best classification accuracy in order to integrate it in our mobile system. The advantage of machine learning techniques is that they allow an evaluation of the state of voice health using simultaneously all four considered acoustic parameters, namely, F_0_, jitter, shimmer, and HNR. Additionally, these techniques are able to classify a voice sample as healthy or pathological analyzing only these parameters and not considering a range of variability. In doing so, they remove the influence of the choice of an appropriate range on the classification accuracy, choice not easy to make, given the lack of a standard range of variability where the acoustic parameters can be evaluated as healthy or pathological.

During the last few years, several methodologies based on machine learning techniques have been used in many biomedical applications [34]. These techniques are capable of learning and/or adapting their structure to the observed data to optimize the classification of a sample. Evaluating a set of data, their aim is to build a model, which approximates, according to the values assumed by independent variables corresponding to the measurable characteristics of each sample, the so-called* features*, the values assumed by a dependent variable, corresponding to a characteristic of interest, the* class,* available for a set of samples necessary for the learning of the model.

In literature various studies exist, which classify the voice signal as pathological or healthy [35–37]. In this study, several machine learning techniques were used to evaluate the capability of each parameter to classify a voice as pathological or healthy. The algorithms employed include the Support Vector Machine (SVM) [38], one of the main techniques used in the literature, and Decision Tree (DT) [39], an algorithm easy to interpret where the Decision Tree represents the learned function. The tree structure is combined with logistic regression models in the Logistic Model Tree (LMT) classifier [40]. The other techniques evaluated are Bayesian classifier (BC) [41] that estimates the probabilistic model that represents a set of random variables and their conditional dependencies and the Instance-based Learning (Ibk) algorithm that achieves a classification through specific instances [42].

In detail, we have the following:

- SVM [38]: this is a classifier where data belonging to different classes are divided by a separating hyperplane. The identification of the class of belonging of several data is the aim of this algorithm, identifying the optimal hyperplane, equally distant from the support vectors from different classes. The optimal hyperplane is selected to maximize the margin, that is, the distance between the hyperplane and its support vectors.
- DT [39]: this is a hierarchical model for supervised learning, composed of internal decision nodes and terminal leaves. The branches are labeled with discrete outcomes of function that each decision node implements. Given an input, a test is applied at each node and one of the branches is considered depending on the outcome.
- LMT [40]: this consists of a Decision Tree structure with logistic regression functions at the leaves. Unlike ordinary decision trees, these leaves have not associated a class label, but a logistic regression function.
- BC [41]: this uses a probabilistic model for the classification, where a set of random variables and their conditional dependences are, respectively, represented as nodes and strings.
- Ibk [42]: this implements the K-nearest neighbors classifier. This is based on the concept that the instances of a set of data that share certain characteristics generally appear to be near in the multidimensional space. The classification of a new element is carried out by looking for the class that appears with the largest number of times in all its k neighbors.

All the analyses have been performed using the WEKA [43] tool, one of the most commonly used tools for data mining tasks due to its efficiency, flexibility, and accessibility.

*Machine Learning Analysis Results. *For each technique, a 10-fold cross-validation was used to evaluate the considered classifiers. The achieved results are shown in Table 13.

Comparing the accuracy obtained using the machine learning classifiers considered, Vox4Health achieves better results than Praat, with a great difference in some cases, such as with BC and DT. The best performance was achieved by using the LMT classifier. The obtained accuracy is equal to 77.4%, while the sensitivity is about 95% and specificity is 31%. These last two values shown are not the best obtained by observing the different ML techniques, but LMT is the technique able to offer the best proportion between the two.

Also in this case we have analyzed the performance for each category of pathology: hyperkinetic and hypokinetic dysphonia and reflux laryngitis, as shown in Tables 14, 15, and 16.

The results obtained indicate that our proposed system provides a good estimation of the acoustic parameters considered, being able to classify correctly voices as healthy or pathological. Reflux laryngitis and hyperkinetic dysphonia are the pathologies better detected, considering the performances of the main machine learning techniques, in particular of the Decision Tree algorithm and Bayesian classifier.

Observing the results obtained, we can note that there was an improvement of classification accuracy using the ML algorithms rather than the rule-base analysis. In particular, the Logistic Model Tree (LMT) achieved an accuracy of about 77% while the best accuracy obtained with single acoustic parameters was achieved by jitter and shimmer (72%). There were improvements also in the specificity percentages (31% for LMT versus 12 % for jitter and 29% for shimmer). This increase of specificity was remarked observing the performance for each category of pathology. Decision Tree achieved a specificity of about 81% for voices suffering from hyperkinetic dysphonia while the Bayesian classifier achieved specificities equal to 98.3% and 89.6%, respectively, for voices suffering from hypokinetic dysphonia and reflux laryngitis.

## 7. Conclusions

Voice disorders can have a significant negative impact on the social and professional life of those afflicted. Although such disorders are often underestimated, their early detection and accurate diagnosis are necessary to reduce serious consequences. Computer-based systems, such as m-health solutions, provide an opportunity to improve and support the main medical techniques necessary to diagnose the presence of these disorders. Vox4Health is an m-health solution, able to estimate in real time the characteristic parameters of the acoustic analysis. This is an important medical examination, useful for the quantitative characterization of vocal dysfunctions.

In this paper, we have presented a clinical study conducted to evaluate the classification accuracy of the proposed m-health solution in assessing a voice as healthy or pathological, comparing the performances obtained with Praat, one of the most commonly used tools for voice analysis in clinical practice. Using a rule-base algorithm to classify a voice as healthy or pathological, the results show that Vox4Health is more effective in identifying the presence of a pathological voice when a pathology is indeed present than Praat, in particular observing the performance of jitter and shimmer. This result is not confirmed when a healthy voice is evaluated. To improve this, we have tested several machine learning techniques to evaluate the state of voice health. In fact, the Logistic Model Tree (LMT) achieved an accuracy of about 77% while the best accuracy obtained with single acoustic parameters was achieved by jitter and shimmer (72%). There were improvements also in the specificity percentages (31% for LMT versus 12 % for jitter and 29% for shimmer). This increase of specificity was remarked observing the performance for each category of pathology. Decision Tree achieved a specificity of about 81% for voices suffering from hyperkinetic dysphonia while the Bayesian classifier achieved specificities equal to 98.3% and 89.6%, respectively, for voices suffering from hypokinetic dysphonia and reflux laryngitis.

It should be noted that the developed app can be used for a first screening test but does not provide a diagnosis. The aim is to evaluate the potential presence of a voice alteration, an index of a disease of the pneumophonic apparatus, and to suggest a consultation with a medical specialist for an accurate voice control. In fact, our analysis on our mobile solution is limited to the performance of the acoustic analysis and the completion of two self-perception questionnaires (VHI and RSI). Other examinations are necessary to perform a correct diagnosis of a voice disorder, such as laryngoscopy, an invasive examination useful to observe the vocal folds and their possible alterations that only a medical specialist can perform. Moreover, Vox4Health does not evaluate all eleven acoustic parameters provided for in the SIFEL protocol. In this first version, we evaluate only four of these: F_0_, jitter, shimmer, and HNR. These are the most useful and significant to detect laryngeal alterations and, for this reason, the ones principally analyzed in clinical practice, as suggested by the medical specialists involved in the project.

## Figures and Tables

**Figure 2:**
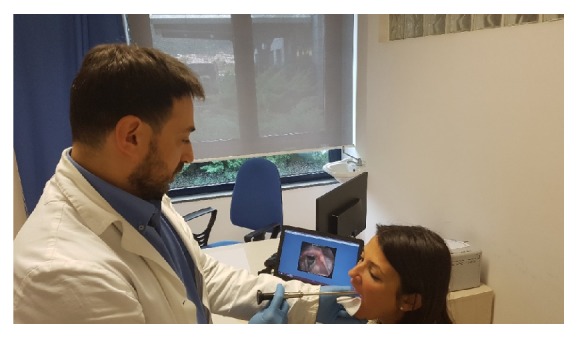
Representation of a laryngoscopic examination performed during the clinical study.

**Table 1:** Procedures for the evaluation of Vox4Health.

Phase	Description
Recruitment	The recruitment of the participants was performed by means of publicizing of the trial process, involving a suitable number of people, through information campaigns, using posters, brochures and meetings. Subjects who met the criteria for inclusion were invited to participate in the process.

Registration of participants	In this phase three documents were delivered to the participants. The first one was the information sheet to indicate the aim of the study, the procedures involved, the benefits and the possible risks of the examinations. Additionally, two forms were signed by the participants, the informed consent and the authorization for the processing of personal data, in order to protect the participants' privacy.

Medical phoniatric examinations	All recruited subjects were examined by medical experts involved in the study to diagnose the presence or not of a voice disorder. In accordance with the guidelines of the SIFEL protocol, they performed a scrupulous anamnesis to collect information relating to lifestyle (for example smoking status, alcohol consumption, voice use and professional activity) and previous or concomitant diseases, such as gastro-esophageal reflux, together with other data of interest to perform a correct diagnosis. Subsequently, the specialists performed a laryngoscopy, an invasive examination necessary to investigate the anatomical structure and any possible alterations of the larynx [44–46]. Figure 2 shows an image of a doctor performing this examination. The instruments employed were a Storz Laryngoscope, the 6.0 mm autoclavable 70° stiff model, used in most cases, and the 2.8 mm flexible model to perform laryngoscopies via the nose for more sensible subjects. Finally, the participants were invited to compile two self-assessment questionnaires required by the SIFEL protocol, the Voice Handicap Index (VHI) [47] and Reflux Symptom Index (RSI) [48], to evaluate, respectively, the psycho-social consequences of any voice disorders and the presence of gastro-esophageal reflux.

Vox4Health testing	The tests, conducted individually, were carried out in a quiet room (<30 dB of background noise) which was not too dry (humidity greater than 30-40%). The subjects recorded the vowel /a/ with a constant intensity, having been trained two or three times before the vocal acquisition. After the audio capture the estimation of each acoustic parameter was shown. The app was installed on a Samsung Galaxy S4, Android version 5.0.1. The distance between the microphone of the mobile device and patient was about 20 cm with an inclination angle of about 45°. All the voice samples were saved in Wave files, sampled at 8000 Hz, and their resolution was 32 bits, recorded in a mono-channel format.

**Table 2:** Details of the voice signals collected in the study.

**Category**	**Gender**	**Number**	%
Healthy	Female	37	17.8%
	Male	21	10.1%

Pathological	Female	47	22.6%
*Hyperkinetic*	Male	23	11.1%
*dysphonia*			

Pathological	Female	32	15.4%
*Hypokinetic*	Male	9	4.3%
*Dysphonia*			

Pathological	Female	19	9.1%
*Laryngitis*	Male	20	9.6%
*Reflux*			

***Total***	***Female***	***135***	***64.9***%
	***Male***	***73***	***35.1***%

**Table 3:** VHI obtained results.

**VHI Score**	**Category**	**Number**	%
0 ≤ VHI ≤ 32	No perceived voice disorder	155	74.5 %

33 ≤ VHI ≤ 43	Mild perceived voice disorder	19	9.1 %

44 ≤ VHI ≤ 60	Moderate perceived voice disorder	16	7.7 %

61 ≤ VHI ≤ 180	Severe perceived voice disorder	18	8.7 %

**Table 4:** RSI obtained results.

**RSI Score**	**Category**	**Number**	%
0 ≤ RSI ≤ 12	No perceived laryngopharyngeal disorder	112	53.8 %

13 ≤ RSI ≤ 45	Perceived laryngopharyngeal disorder	96	46.2 %

**Table 5:** Healthy range for the acoustic parameters considered.

Parameter	Gender	Healthy range
F_0_ (Hz)	Female	189-280 (Hz)
	Male	104-158 (Hz)
Jitter (%)	Female	<1.04 %
	Male	<1.04 %
Shimmer (dB)	Female	<0.35 dB
	Male	<0.35 dB
HNR (dB)	Female	>20 dB
	Male	>20 dB

**Table 6:** Results about the reliability of the measurements.

**Voice ID**	**Gender**	**Diagnosis of the Medical Expert**	**F**_**0**_	**Classification based on F**_**0**_	**jitter**	**Classification based on jitter**	**shimmer**	**Classification based on shimmer**	**HNR**	**Classification based on HNR**	**Note**
voice 061	f	Healthy	217	Healthy	1.323	Pathological	1.520	Pathological	24.36	Healthy	
			211	Healthy	1.323	Pathological	1.518	Pathological	26.985	Healthy	

voice 076	f	Pathological	212	Healthy	1.306	Pathological	2.565	Pathological	4.302	Pathological	File Corrupted - the vocalization
			320	Pathological	1.318	Pathological	2.344	Pathological	7.738	Pathological	is less than 5 seconds

voice 089	f	Pathological	148	Pathological	1.140	Pathological	1.392	Pathological	0.457	Pathological	
			154	Pathological	1.322	Pathological	2.503	Pathological	1.915	Pathological	

voice 108	m	Healthy	188	Pathological	1.003	Healthy	0.024	Healthy	24.749	Healthy	File Corrupted - the user speaks
			153	Healthy	1.323	Pathological	3.045	Pathological	8.486	Pathological	during the vocalization

voice 125	f	Pathological	40	Pathological	0.937	Healthy	2.093	Pathological	15.906	Pathological	
			41	Pathological	0.759	Healthy	2.744	Pathological	10.898	Pathological	

voice 128	m	Pathological	175	Pathological	1.305	Pathological	4.214	Pathological	5.465	Pathological	
			160	Pathological	1.363	Pathological	1.885	Pathological	5.125	Pathological	

voice 138	m	Pathological	251	Pathological	1.306	Pathological	1.747	Pathological	11.995	Pathological	
			229	Pathological	1.306	Pathological	1.733	Pathological	10.626	Pathological	

voice 146	f	Healthy	145	Pathological	0.878	Healthy	0.025	Healthy	6.048	Pathological	
			157	Pathological	0.671	Healthy	0.206	Healthy	2.481	Pathological	

voice 147	m	Pathological	215	Pathological	1.306	Pathological	3.903	Pathological	6.217	Pathological	
			222	Pathological	1.288	Pathological	2.0585	Pathological	5.836	Pathological	

voice 167	f	Pathological	325	Pathological	1.075	Pathological	3.861	Pathological	0.783	Pathological	
			305	Pathological	1.065	Pathological	1.037	Pathological	2.518	Pathological	

voice 169	m	Pathological	647	Pathological	1.306	Pathological	2.453	Pathological	13.033	Pathological	
			603	Pathological	1.306	Pathological	1.363	Pathological	13.625	Pathological	

voice 179	f	Healthy	239	Healthy	1.306	Pathological	7.959	Pathological	13.551	Pathological	
			237	Healthy	1.306	Pathological	1.844	Pathological	17.641	Pathological	

voice 182	f	Healthy	215	Healthy	1.306	Pathological	2.779	Pathological	22.578	Healthy	
			216	Healthy	1.306	Pathological	6.293	Pathological	25.158	Healthy	

voice 188	f	Pathological	261	Pathological	0.901	Healthy	0.107	Healthy	9.105	Pathological	File Corrupted - the user coughs
			250	Healthy	1.247	Pathological	1.272	Pathological	11.219	Pathological	during the vocalization

voice 193	m	Pathological	377	Pathological	1.061	Pathological	0.367	Pathological	15.522	Pathological	
			356	Pathological	1.321	Pathological	2.803	Pathological	14.917	Pathological	

voice 194	f	Pathological	524	Pathological	1.306	Pathological	3.466	Pathological	0.682	Pathological	
			505	Pathological	1.279	Pathological	2.549	Pathological	0.821	Pathological	

voice 197	f	Healthy	225	Healthy	1.305	Pathological	2.153	Pathological	24.895	Healthy	
			212	Healthy	1.259	Pathological	1.842	Pathological	21.939	Healthy	

voice 203	f	Pathological	135	Pathological	1.154	Pathological	1.929	Pathological	11.343	Pathological	
			119	Pathological	1.046	Pathological	0.016	Healthy	14.344	Pathological	

**Table 7:** Results of some pathological voices about the portability of the Vox4Health app among the different Android devices tested.

**Voice ID**		**F**_**0**_	**Classification** **based on F**_**0**_	**jitter**	**Classification** **based on** **jitter**	**shimmer**	**Classification** **based on** **shimmer**	**HNR**	**Classification** **based on** **HNR**
voice 003	Samsung S4	244	Pathological	1.26	Pathological	2.1	Pathological	22.64	Healthy
m	Huawei	203	Pathological	1.27	Pathological	2.01	Pathological	20.5	Healthy
	Asus	189	Pathological	1.26	Pathological	1.99	Pathological	25.39	Healthy
	One Plus	210	Pathological	1.17	Pathological	2.01	Pathological	22.22	Healthy
	**DEV.ST**	**23.3**		**0.05**		**0.05**		**2.03**	

voice 026	Samsung S4	247	Pathological	1.21	Pathological	1.28	Pathological	18.25	Pathological
m	Huawei	221	Pathological	1.2	Pathological	1.26	Pathological	19.86	Pathological
	Asus	276	Pathological	1.19	Pathological	1.24	Pathological	17.89	Pathological
	One Plus	236	Pathological	1.19	Pathological	1.29	Pathological	18.31	Pathological
	**DEV.ST**	**23.25**		**0.01**		**0.01**		**0.87**	

voice 041	Samsung S4	225	Pathological	1.18	Pathological	0.95	Pathological	24.34	Healthy
m	Huawei	203	Pathological	1.19	Pathological	0.96	Pathological	20.44	Healthy
	Asus	192	Pathological	1.19	Pathological	0.95	Pathological	22.17	Healthy
	One Plus	189	Pathological	1.19	Pathological	0.98	Pathological	22.44	Healthy
	**DEV.ST**	**16.32**		**0.01**		**0.01**		**1.60**	

voice 047	Samsung S4	228	Pathological	1.18	Pathological	2.02	Pathological	16.82	Pathological
m	Huawei	189	Pathological	1.17	Pathological	1.99	Pathological	14.78	Pathological
	Asus	220	Pathological	1.19	Pathological	1.99	Pathological	13.1763	Pathological
	One Plus	209	Pathological	1.17	Pathological	2.01	Pathological	12.82	Pathological
	**DEV.ST**	**16.90**		**0.01**		**0.02**		**1.83**	

voice 055	Samsung S4	367	Pathological	1.06	Pathological	0.7	Pathological	26.69	Healthy
m	Huawei	357	Pathological	1.18	Pathological	0.72	Pathological	24.4	Healthy
	Asus	339	Pathological	1.06	Pathological	0.71	Pathological	23.77	Healthy
	One Plus	335	Pathological	1.14	Pathological	0.75	Pathological	22.12	Healthy
	**DEV.ST**	**15.09**		**0.06**		**0.02**		**1.89**	

voice 084	Samsung S4	207	Healthy	1.21	Pathological	1.63	Pathological	15.39	Pathological
f	Huawei	222	Healthy	1.19	Pathological	1.64	Pathological	12.59	Pathological
	Asus	229	Healthy	1.19	Pathological	1.6	Pathological	18.61	Pathological
	One Plus	237	Healthy	1.18	Pathological	1.59	Pathological	14.13	Pathological
	**DEV.ST**	**12.74**		**0.01**		**0.02**		**2.56**	

voice 086	Samsung S4	285	Pathological	1.15	Pathological	0.91	Pathological	30.28	Healthy
f	Huawei	286	Pathological	1.19	Pathological	0.92	Pathological	32.l	Healthy
	Asus	277	Pathological	1.3	Pathological	0.95	Pathological	33.23	Healthy
	One Plus	303	Pathological	1.18	Pathological	0.97	Pathological	33.9	Healthy
	**DEV.ST**	**10.94**		**0.07**		**0.03**		**1.58**	

voice 102	Samsung S4	238	Healthy	1.28	Pathological	0.76	Pathological	25.51	Healthy
f	Huawei	241	Healthy	1.31	Pathological	0.73	Pathological	22,61	Healthy
	Asus	211	Healthy	1.27	Pathological	0.74	Pathological	22.26	Healthy
	One Plus	243	Healthy	1.3	Pathological	0.77	Pathological	24.94	Healthy
	**DEV.ST**	**14.97**		**0.02**		**0.02**		**1.63**	

voice 202	Samsung S4	284	Pathological	1.15	Pathological	1.84	Pathological	21.12	Healthy
f	Huawei	296	Pathological	1.06	Pathological	1.86	Pathological	22.93	Healthy
	Asus	303	Pathological	1.15	Pathological	1.89	Pathological	20.11	Healthy
	One Plus	296	Pathological	1.11	Pathological	1.84	Pathological	22.84	Healthy
	**DEV.ST**	**7.89**		**0.04**		**0.02**		**1.37**	

voice 208	Samsung S4	234	Healthy	1.2	Pathological	1.14	Pathological	21.949	Healthy
f	Huawei	238	Healthy	1.19	Pathological	1.16	Pathological	20.8	Healthy
	Asus	212	Healthy	1.19	Pathological	1.17	Pathological	24.79	Healthy
	One Plus	196	Healthy	1.13	Pathological	1.12	Pathological	21.78	Healthy
	**DEV.ST**	**19.66**		**0.03**		**0.02**		**1.72**	

**Table 8:** Results of some healthy voices about the portability of the Vox4Health app among the different Android devices tested.

**Voice ID**		**F**_**0**_	**Classification based on F**_**0**_	**jitter**	**Classification based on jitter**	**shimmer**	**Classification** **based on** **shimmer**	**HNR**	**Classification** **based on** **HNR**
voice 045	Samsung S4	233	Healthy	1.2	Pathological	0.18	Healthy	21.95	Healthy
f	Huawei	237	Healthy	1.21	Pathological	0.22	Healthy	21.00	Healthy
	Asus	254	Healthy	1.19	Pathological	0.19	Healthy	21.89	Healthy
	One Plus	209	Healthy	1.12	Pathological	0.16	Healthy	20.36	Healthy
	**DEV.ST**	**18.55**		**0.04**		**0.02**		**0.76**	

voice 095	Samsung S4	375	Pathological	1.21	Pathological	1.4	Pathological	28.91	Healthy
m	Huawei	368	Pathological	1.16	Pathological	1.45	Pathological	32.91	Healthy
	Asus	302	Pathological	1.19	Pathological	1.47	Pathological	32.00	Healthy
	One Plus	359	Pathological	1.17	Pathological	1.48	Pathological	30.28	Healthy
	**DEV.ST**	**33.32**		**0.02**		**0.04**		**1.78**	

voice 097	Samsung S4	210	Healthy	1.2	Pathological	2.56	Pathological	28.19	Healthy
f	Huawei	223	Healthy	1.2	Pathological	2.58	Pathological	33.00	Healthy
	Asus	227	Healthy	1.19	Pathological	2.57	Pathological	31.32	Healthy
	One Plus	231	Healthy	1.15	Pathological	2.5	Pathological	30.2	Healthy
	**DEV.ST**	**9.11**		**0.02**		**0.04**		**2.02**	

voice 100	Samsung S4	148	Healthy	1.2	Pathological	1.04	Pathological	14.80	Pathological
m	Huawei	107	Healthy	1.22	Pathological	1.05	Pathological	12.01	Pathological
	Asus	126	Healthy	1.19	Pathological	1.01	Pathological	14	Pathological
	One Plus	127	Healthy	1.15	Pathological	1.09	Pathological	19.51	Pathological
	**DEV.ST**	**16.75**		**0.03**		**0.03**		**3.18**	

voice 104	Samsung S4	133	Healthy	1.2	Pathological	1.,5	Pathological	21.67	Healthy
m	Huawei	119	Healthy	1.2	Pathological	1.06	Pathological	21.07	Healthy
	Asus	120	Healthy	1.19	Pathological	1.03	Pathological	23.17	Healthy
	One Plus	150	Healthy	1.17	Pathological	1.01	Pathological	20.48	Healthy
	**DEV.ST**	**14.48**		**0.01**		**0.02**		**1.15**	

voice 107	Samsung S4	109	Healthy	1.06	Pathological	2.37	Pathological	25.37	Healthy
m	Huawei	128	Healthy	1.11	Pathological	2.38	Pathological	24.03	Healthy
	Asus	136	Healthy	1.06	Pathological	2.3	Pathological	24.99	Healthy
	One Plus	140	Healthy	1.07	Pathological	2.41	Pathological	24.16	Healthy
	**DEV.ST**	**13.77**		**0.02**		**0.05**		**0.65**	

voice 109	Samsung S4	304	Pathological	1.2	Pathological	0.37	Pathological	30.47	Healthy
f	Huawei	311	Pathological	1.2	Pathological	0.43	Pathological	28.6	Healthy
	Asus	276	Pathological	1.22	Pathological	0.45	Pathological	31.04	Healthy
	One Plus	322	Pathological	1.21	Pathological	0.39	Pathological	27.35	Healthy
	**DEV.ST**	**19.62**		**0.01**		**0.04**		**1.70**	

voice 123	Samsung S4	211	Healthy	1.08	Pathological	0.2	Healthy	29.87	Healthy
f	Huawei	245	Healthy	1.07	Pathological	0.24	Healthy	32	Healthy
	Asus	242	Healthy	1.06	Pathological	0.19	Healthy	29.97	Healthy
	One Plus	222	Healthy	1.05	Pathological	0.17	Healthy	32.09	Healthy
	**DEV.ST**	**16.27**		**0.01**		**0.03**		**1.23**	

voice 158	Samsung S4	181	Pathological	1.23	Pathological	1.02	Pathological	33.99	Healthy
m	Huawei	206	Pathological	1.2	Pathological	1.09	Pathological	32.12	Healthy
	Asus	181	Pathological	1.19	Pathological	1.03	Pathological	33.58	Healthy
	One Plus	171	Pathological	1.17	Pathological	1.04	Pathological	32.76	Healthy
	**DEV.ST**	**14.93**		**0.03**		**0.03**		**0.084**	

voice 180	Samsung S4	224	Healthy	1.13	Pathological	0.66	Pathological	23.35	Healthy
f	Huawei	209	Healthy	1.18	Pathological	0.55	Pathological	21	Healthy
	Asus	239	Healthy	1.19	Pathological	0.67	Pathological	22.26	Healthy
	One Plus	241	Healthy	1.19	Pathological	0.62	Pathological	21.92	Healthy
	**DEV.ST**	**14.91**		**0.03**		**0.02**		**0.97**	

**Table 9:** Classification results obtained with Vox4Health and Praat.

Parameter	System	Accuracy (%)	Sensitivity (%)	Specificity (%)
F_0_ (Hz)	Vox4Health	**54.3**	**53.3**	56.9
	Praat	50.0	44.0	**65.5**
Jitter (%)	Vox4Health	**72.6**	**96.0**	12.1
	Praat	40.4	20.0	**93.1**
Shimmer (dB)	Vox4Health	**72.6**	**89.3**	29.3
	Praat	57.7	58.7	**55.2**
HNR (dB)	Vox4Health	61.1	64.7	**51.7**
	Praat	**63.0**	**71.3**	41.4

**Table 10:** Classification results obtained for subjects suffering from hyperkinetic dysphonia.

Parameter	System	Accuracy (%)	Sensitivity (%)	Specificity (%)
F_0_ (Hz)	Vox4Health	**58.2**	**59.4**	56.9
	Praat	54.7	45.7	**65.5**
Jitter (%)	Vox4Health	**59.4**	**98.6**	12.1
	Praat	48.4	12.9	**91.4**
Shimmer (dB)	Vox4Health	**62.5**	**90.0**	29.3
	Praat	57.0	58.6	**55.2**
HNR (dB)	Vox4Health	**63.6**	**73.2**	**51.8**
	Praat	55.5	67.1	41.4

**Table 11:** Classification results obtained for subjects suffering from hypokinetic dysphonia.

Parameter	System	Accuracy (%)	Sensitivity (%)	Specificity (%)
F_0_ (Hz)	Vox4Health	**57.6**	**58.6**	56.9
	Praat	57.6	46.3	**65.5**
Jitter (%)	Vox4Health	43.4	**87.8**	12.1
	Praat	**72.7**	46.3	**91.4**
Shimmer (dB)	Vox4Health	54.5	**90.2**	29.3
	Praat	**67.7**	85.3	**55.2**
HNR (dB)	Vox4Health	55.6	61.0	**51.7**
	Praat	**61.6**	**90.2**	41.4

**Table 12:** Classification results obtained for subjects suffering from reflux laryngitis.

Parameter	System	Accuracy (%)	Sensitivity (%)	Specificity (%)
F_0_ (Hz)	Vox4Health	52.6	**46.2**	56.9
	Praat	**54.6**	38.5	**65.5**
Jitter (%)	Vox4Health	45.1	**88.6**	12.1
	Praat	**55.7**	2.6	**91.4**
Shimmer (dB)	Vox4Health	**52.6**	**87.2**	29.3
	Praat	45.4	30.7	**55.2**
HNR (dB)	Vox4Health	**51.5**	51.3	**51.8**
	Praat	48.4	**58.9**	41.4

**Table 13:** Classification results obtained by using machine-learning techniques over a database containing all parameters.

Machine Learning Techniques	System	Accuracy (%)	Sensitivity (%)	Specificity (%)
SVM	Vox4Health	72.6	**99.3**	3.4
	Praat	71.6	99.3	0
DT	Vox4Health	75.5	96.7	20.7
	Praat	69.7	85.3	29.3
BC	Vox4Health	76.0	95.3	26.0
	Praat	67.3	86.0	19.0
LMT	Vox4Health	**77.4**	95.3	31.0
	Praat	76.9	94.7	31.0
Ibk	Vox4Health	63.9	74.7	**36.2**
	Praat	63.9	73.3	39.7

**Table 14:** Classification results obtained by using machine-learning techniques over a database containing all parameters for subjects suffering from hyperkinetic dysphonia.

Machine Learning Techniques	System	Accuracy (%)	Sensitivity (%)	Specificity (%)
SVM	Vox4Health	62.5	56.9	67.1
	Praat	63.3	58.6	67.1
DT	Vox4Health	**66.4**	48.3	**81.4**
	Praat	53.9	75.7	27.6
BC	Vox4Health	58.6	**93.1**	30
	Praat	50.0	90.0	1.7
LMT	Vox4Health	64.1	55.2	71.4
	Praat	63.3	64.3	62.1
Ibk	Vox4Health	56.2	46.5	64.3
	Praat	56.2	64.3	46.5

**Table 15:** Classification results obtained by using machine-learning techniques over a database containing all parameters for subjects suffering from hypokinetic dysphonia.

Machine Learning Techniques	System	Accuracy (%)	Sensitivity (%)	Specificity (%)
SVM	Vox4Health	64.4	46.4	77.6
	Praat	73.7	48.8	91.4
DT	Vox4Health	61.6	53.6	67.2
	Praat	63.6	68.3	60.3
BC	Vox4Health	66.7	21.9	**98.3**
	Praat	**73.7**	**70.7**	75.9
LMT	Vox4Health	60.6	43.9	72.4
	Praat	72.7	53.6	86.2
Ibk	Vox4Health	53.5	43.9	60.3
	Praat	59.6	58.5	60.3

**Table 16:** Classification results obtained by using machine-learning techniques over a database containing all parameters for subjects suffering from reflux laryngitis.

Machine Learning Techniques	System	Accuracy (%)	Sensitivity (%)	Specificity (%)
SVM	Vox4Health	**73.2**	56.4	84.5
	Praat	68.0	51.3	79.3
DT	Vox4Health	64.9	71.8	60.3
	Praat	61.8	84.6	46.5
BC	Vox4Health	62.9	23.1	**89.6**
	Praat	64.9	**100**	41.4
LMT	Vox4Health	61.8	53.8	67.2
	Praat	63.9	51.3	72.4
Ibk	Vox4Health	68.0	66.7	69.0
	Praat	67.0	59.0	72.4

## Data Availability

The collected data is available at physionet (https://physionet.org/pn6/voiced/).
